# Analysis of the 2013-2020 WHO Global Action Plan for the Prevention and Control of Noncommunicable Diseases Using the 2008 Davies Provenance Framework​

**DOI:** 10.7759/cureus.115666

**Published:** 2026-09-02

**Authors:** Ikenna Joel Edward, Pamela Momah

**Affiliations:** 1 Medicine, University Hospitals of Morecambe Bay NHS Foundation Trust, Kendal, GBR; 2 Orthopedics, University of Chester, Chester, GBR

**Keywords:** global health, noncommunicable disease (ncds), policy analysis, public health policy, world health organization (who)

## Abstract

Noncommunicable diseases (NCDs) account for 41 million deaths annually, making them the world’s leading cause of death. To address this growing crisis, the World Health Organization (WHO) introduced and implemented the Global Action Plan for the Prevention and Control of Noncommunicable Diseases 2013-2020. In this plan, the WHO provided a strategic roadmap to achieve a relative reduction of 25% in premature NCD deaths by 2025.

By utilizing and adapting the 2008 Davies Provenance Framework, this review critically evaluated the WHO Global Action Plan. This framework, a policy analysis tool, was adopted to systematically assess the action plan’s origins, priorities, stakeholder involvement, implementation procedures, and potential perversities.

The action plan successfully called for multisectoral collaboration, from the national to the global level, and empowered WHO Member States to adapt its implementation to their unique national circumstances. This analysis revealed anticipated structural resistance arising from competing national development priorities and private-sector interests, particularly those of the alcohol and tobacco industries.

The WHO Global Action Plan offers an extensive and well-structured basis for reducing the global burden of NCDs. However, its success depends on its joint adoption by all stakeholders and on having transformational and transactional leaders champion the drive for behavioral change while addressing unintended perversities.

## Introduction and background

The leading causes of mortality worldwide are noncommunicable diseases (NCDs), namely, diabetes, cancer, chronic obstructive pulmonary disease (COPD), and cardiovascular diseases (CVDs) [1]. NCDs account for 41 million deaths annually, representing 74% of all deaths worldwide [1]. Additionally, 17 million deaths from NCDs occur before the age of 70, and 86% of these premature deaths occur in low- and middle-income countries (LMICs) [1].

Since NCDs are currently the subject of extensive research and understanding, all WHO Member States are well positioned to take appropriate action [2]. Evidence has shown that NCDs are strategically linked to the Sustainable Development Goals (SDGs) and pose a threat to their achievement by hindering economic development, contributing to poverty, and increasing socioeconomic disparities within and between countries [1]. These notable impacts have led to an urgent need to address the issue of NCDs [3].

Although it has been demonstrated that the prevention and control of NCDs can significantly reduce their burden, the health sector or an individual country cannot address this challenge alone [1,4]. This action plan serves as a roadmap containing policy options for all stakeholders, including Member States, to adopt a coordinated approach to addressing NCDs [3]. It prioritizes the reduction of modifiable risk factors common to NCDs, such as smoking, harmful alcohol use, sedentary lifestyles, diabetes, and obesity; strengthening national capacity, healthcare systems, and partnerships; raising awareness; promoting evidence-based research; and monitoring NCD trends, to address NCDs while fostering the achievement of the SDGs.

Public policy is defined as regulations, laws, or actions established by the government to address an issue that has been identified or framed as a problem by either the public or policymakers [5]. It can also be explained as the conversion of government priorities and principles into a series of programs and actions intended to deliver desirable change [5]. Policy process, on the other hand, is a model of decision-making viewed as a sequence of steps that follows a cyclical pattern [6]. The stages of the policy process include problem emergence, agenda setting, consideration of policy options, decision-making, policy implementation, and evaluation [7]. Consistently, the policy process can also be summarized as agenda setting (problem identification), policy formulation (consideration of policy options and decision-making), policy implementation, and policy review (evaluation, monitoring, and reporting) [8].

Additionally, public policy involves ethical judgment, either directly or indirectly, because every stage of the policy cycle has an impact on individuals or society [9]. For this reason, every step of the policy process must be balanced against social and individual values, analyzed to determine how policymakers or the government arrived at a particular problem definition or proposed solution, and assessed to ascertain whether the decision was based on evidence [5,9].

## Review

Objective

The objective of this study is to critically analyze the WHO Global Action Plan for the Prevention and Control of Noncommunicable Diseases 2013-2020 using the Davies (2008) Provenance Framework. This framework was carefully selected over alternative policy analysis tools because it emphasizes stakeholder involvement and operational realities rather than political and policy dimensions [10,11]. 

Methodology

This study employed a retrospective qualitative analysis that systematically mapped a policy document against a predetermined theoretical framework, namely, the Davies Provenance Framework. The WHO Global Action Plan for the Prevention and Control of Noncommunicable Diseases 2013-2020 was independently analyzed by two reviewers. All coding discrepancies were reconciled through meetings and discussion between the reviewers.

The eight domains of the Davies Provenance Framework were adopted as the analytical framework for this analysis. These domains include primary purpose, precedents, priorities, participants, processes and procedures, practicalities, perversities, and perspectives [10].

*Stages of Analysis* 

To reduce subjective bias and ensure analytical rigor, the action plan and supplementary literature were independently analyzed by two reviewers. Each reviewer conducted an in-depth reading of the texts, extracted relevant data, and coded the data according to the categories of the Davies Provenance Framework.

At the end of the coding phase, the analytical matrices were compared by the reviewers. Although there was a high degree of initial agreement, some coding disagreements arose, particularly because of the interrelated nature of the Davies domains. For example, there were discrepancies regarding whether industrial pushback should be coded under practicalities or perversities. Similarly, interpretative overlaps occurred when distinguishing between perspectives and precedents.

Coding disagreements were resolved through discussion and consensus meetings. During these meetings, both reviewers revisited the foundational definitions and meanings established by Davies. The extracted data were then discussed and evaluated until a consensus regarding their categorization was reached.

It is worth noting that statistical calculations were not utilized during the analysis because this study employed a qualitative policy analysis rather than a quantitative measurement approach. Instead, complete agreement (100%) was reached through deliberation, ensuring that the final mapping reflected a balanced, mutually vetted, and rigorous interpretation of the action plan document.

*Theoretical Integration* 

Organizational theories were systematically integrated to help operationalize the Davies domains and ensure methodological rigor. To assess priorities (agenda setting), Kotter’s Change Model and Kingdon’s Multiple Streams Framework were utilized. Arnstein’s Ladder of Participation was applied to understand stakeholder involvement (participants), while Tuckman’s Group Development Model and Lewin’s Force Field Analysis were used to assess potential implementation resistance (practicalities). 

Supplementary Evidence

To support the application of the framework, a supplementary narrative literature search was conducted to identify relevant contextual evidence on NCD epidemiology, socioeconomic determinants, change management, and the unintended consequences of tobacco control. Databases and repositories, including PubMed, WHO, and UN sources, were searched for peer-reviewed articles and official reports using keywords such as “NCD policy implementation”, “stakeholder participation”, and “tobacco taxation socioeconomic effects”.

Review

*Primary Purpose* 

This action plan aims to implement the General Assembly political declaration on the prevention and control of NCDs [3]. It also calls for multisectoral collaboration and cooperation at the national, regional, and global levels to reduce the burden of four major NCDs, including CVDs, COPD, cancer, and diabetes.

NCDs are the world’s leading causes of death, affecting both developed and developing nations [1]. NCDs make the largest contribution to the global burden of mortality and morbidity and account for approximately 80% of total premature deaths [1,12]. For this reason, the action plan seeks to address the consequences of NCDs, including mortality and morbidity, economic losses, and poverty, by focusing on the reduction of four common risk factors: harmful alcohol use, tobacco consumption, unhealthy dietary habits, and sedentary lifestyles [3].

To address the issue of NCDs, global targets centered on risk factors were established [3]. These targets include reducing risk factors and increasing the availability of affordable healthcare services and NCD treatment. The WHO also highlighted six objectives for Member States to achieve the global targets. These objectives comprise promoting international collaboration and campaigns through national, regional, and global agendas to increase the priority given to the prevention and control of NCDs; improving countries’ responses to NCD prevention and control by building strong partnerships, leadership, and national capacity; and creating environments that promote health and reduce risk factors and social determinants of health [3]. The objectives further include strengthening healthcare systems and adopting people-centered primary healthcare, promoting and supporting research and development at the national level, monitoring NCD trends, and evaluating progress.

Underpinning this action plan is the WHO vision of a world free from the burden of NCDs [3]. Aligning this vision with the policy options embedded in the action plan, which highlight the adoption of policies according to individual nations’ circumstances, it is evident that the vision is consistent with a democratic approach because it recognizes national sovereignty and freedom of choice [3,13].

*Precedents* 

Looking backward helps identify successful frameworks implemented by an organization and provides an opportunity to learn from previous mistakes [14]. Consistent with this approach, the review identified several Health Assembly decisions adopted in support of the core elements of the global plan for the prevention and management of NCDs since its approval in 2000 [3]. This action plan builds on these decisions by further advancing their implementation. Other resolutions on which this action plan was built include the “WHO Framework Convention on Tobacco Control,” “Sustainable Universal Coverage and Health Financing,” “Global Strategy on the Reduction of the Harmful Use of Alcohol,” “Global Plan of Action on Intellectual Property, Public Health and Innovation,” and “Global Strategy on Health, Diet and Physical Activity” [3]. It is also closely conceptualized and strategically aligned with the action plans for the “Prevention of Avoidable Blindness and Visual Impairment 2014-2019” and the “Comprehensive Mental Health Action Plan 2013-2020.”

This action plan was initiated by WHO to mobilize global collaboration and participation in reducing NCDs and their risk factors. It is underpinned by principles and values related to human rights, including the right to the highest attainable standard of health; social justice, including equitable distribution of resources; cooperation and solidarity; and the empowerment of people and communities [3]. Finally, it follows the principles of Universal Health Coverage and a life-course approach, which emphasizes interventions beginning early in life [3].

*Priorities* 

Kotter’s first step in the Change Model posits that successful reform initiatives must begin by creating a sense of urgency. This is achieved by understanding and communicating the pressures that necessitate change [15]. Consistent with this principle, WHO’s efforts to understand and communicate the impact of NCDs created a sense of urgency and pressure for the development of this action plan. NCDs have a broad impact on both individuals and the economy [1,12]. Individuals with NCDs experience financial strain due to increasing healthcare costs and reduced ability to participate in employment [1,12]. Consequently, these circumstances can push families into poverty, which creates a cascade of adverse outcomes, including increased school dropout rates, poor nutrition and food insecurity, inadequate housing, and the adoption of harmful behaviors such as alcohol use and smoking, ultimately increasing social inequalities [16]. Based on this, the broader consequences of poverty can create a vicious cycle that increases exposure to NCD risk factors and, consequently, the burden of NCDs [16]. At the economic level, NCDs are estimated to cause a total output loss of approximately USD 47 trillion, equivalent to about 75% of global GDP. These losses are attributed to increasing healthcare expenditures, absenteeism, and reduced productivity [12].

For the issue of NCDs to enter the government’s political discourse or agenda, the problem, politics, and policy streams must converge through a series of opportunities [17]. The problem stream, represented by the burden of NCDs and the threat they pose to the achievement of the SDGs, created pressure on policymakers to pursue policy change. Additionally, the context surrounding this problem created a window of opportunity by highlighting the need for a comprehensive framework for collaboration, partnership, and leadership at the national, regional, and global levels to prevent and control NCDs. However, governments must tailor the formulation and implementation of these policies to their individual national contexts. Finally, the resolution reached among governments, WHO, and United Nations agencies concerning NCDs resulted in the development of the “Global Action Plan for Prevention and Control of Noncommunicable Diseases 2013-2020.”

The application of Kingdon's model of policy analysis demonstrates the interconnectedness of the three streams and illustrates how the identification of problems can influence policymakers to pursue change and formulate and implement policies.

*Participants* 

For a change process to be successful, a “guiding coalition” of individuals needs to be created, as no single person can effectively lead and manage a change process [18]. This concept is consistent with Kotter’s second stage of the Change Model, which emphasizes the importance of establishing the right “guiding coalition” to champion the change process [15]. Accordingly, to prevent a policy from failing, an appropriate coalition must be identified [19]. These coalitions comprise stakeholders who have an interest in seeing change occur or who are affected by the change, possess positions of power, demonstrate effective leadership, and have relevant expertise and credibility [15]. In contrast, service users are the beneficiaries of an intervention [20]. Based on this definition, service users can be considered stakeholders; however, not all policies recognize service users as stakeholders, and they may consequently be excluded from policy-making processes [20].

Looking upward is essential for identifying which stakeholders are affected by a policy and need to be involved [14]. Consistent with this approach, stakeholders at the national, regional, and global levels were identified. These include WHO Member States, the United Nations, international organizations, public health professionals, academia, private-sector organizations, nongovernmental organizations, professional bodies, financial institutions such as the World Bank, health personnel, individuals, religious institutions, civil society, and relevant government ministries, including those responsible for education, finance, housing, social welfare, employment, and economic development [3].​ 

These stakeholders were engaged in the action plan process through meetings coordinated by WHO regional offices, informal consultations with Member States, web-based consultations, and informal discussions with nongovernmental organizations and private-sector representatives [3]. However, as engagement does not necessarily equate to involvement, it is important to distinguish between stakeholder engagement and meaningful participation in the action plan process [21]. Stakeholder involvement, rather than engagement alone, is essential to ensuring that policies are tailored to needs, designed and implemented effectively, and that resources are appropriately prioritized [22].

In accordance with the stakeholder mapping framework described in [23], WHO leads and coordinates the promotion, monitoring, provision of policy guidance, capacity building, and strengthening of the NCD action plan. However, consistent with the citizen-control level of Arnstein’s Ladder of Participation, national governments hold the primary responsibility for preventing and controlling NCDs [24]. National governments, as representatives of Member States, were delegated the authority and flexibility to adopt the action plan according to their national needs, circumstances, and legislation, while remaining aligned with its six objectives [3]. It is worth noting that, because the action plan represents a global collaboration among WHO Member States, international actors, and private-sector stakeholders, individuals were not identified as citizens within the context of Arnstein’s citizen-control framework; rather, governments occupied this role. Consequently, individuals were not directly represented in the action plan process but were indirectly represented through the national representatives of Member States.

*Process and Procedures* 

For a change to be successfully implemented at the organizational level, time and costs must be clearly defined, and the performance of the team (actors) must be reviewed to determine the type of support or training required [25]. This notion is consistent with Briener’s Performance Management Model, which focuses on looking inward and downward to ascertain the skills, costs, and timeframe required for change [14]. Similarly, Kotter’s fifth step posits that, when empowering employees, obstacles related to organizational structures, skills, systems, and supervision must be addressed [15]. Consistent with these principles, WHO estimated a total implementation cost of USD 940.26 million and a timeframe of 2013-2020 for implementing the action plan [3]. Additionally, because some countries lacked sufficient capacity to implement the action plan, support and training were provided [3]. For example, WHO provided guidance on the measurement, collection, and reporting of target indicators; supported national authorities in strengthening their health systems and improving the quality of services through the development of appropriate infrastructure and training of healthcare personnel; and strengthened national surveillance and monitoring systems to improve NCD data collection [3]. Similarly, national governments, which were tasked with implementing the action plan, provided support in the form of healthcare personnel training, increased investment in infrastructure and NCD treatment, the establishment of public institutions to support NCD monitoring and surveillance, and increased financial allocations for NCD surveillance [3].

*Practicalities* 

Regarding change, it is not uncommon to encounter resistance [22]. As a result, identifying sources of resistance can help determine who supports or opposes a change and facilitate the development of a change matrix to define the influencing activities that may be necessary [22]. Consistent with this perspective, Kurt Lewin posits that, for change to occur, the present condition and the forces maintaining it must be considered [26]. Similarly, Tuckman’s team development model, which comprises the forming, storming, norming, performing, and adjourning/transforming stages, emphasizes the importance of the storming stage, which is characterized by conflict and resistance, in the establishment and adoption of change [27]. Likely areas of resistance to this action plan include the private sector, particularly tobacco and alcohol companies, as well as individuals and national governments [3]. Private companies are profit-oriented organizations and, in some cases, may not be inclined to prioritize ethical or socially responsible behavior when such actions conflict with their commercial interests [28]. In an effort to resist regulation, these companies may conduct campaigns, engage experts and fund scientific research, support legislation promoting voluntary codes, fund political parties, and influence policies through lobbying activities [28]. Similarly, national governments have competing priorities, which may include infrastructure development or addressing specific national disease burdens [29]. This may lead to resistance because funding decisions and policy implementation are often based on national priorities, which, in some developing nations, may place greater emphasis on infrastructure development [29]. In addition, poverty is associated with unhealthy dietary behaviors and increased alcohol and tobacco consumption [30]. Therefore, failure to implement policies that address the effects of poverty may create barriers to the adoption of this action plan among individuals. Other forms of resistance may include protests and media campaigns grounded in human rights and the right to participate in decision-making [28]. 

*Perversities* 

There is an interconnectedness between NCDs and the SDGs [31]. As a result, reducing NCDs can potentially contribute to the achievement of the SDGs [32]. For example, reducing NCDs can promote financial risk protection and reduce poverty by decreasing the catastrophic healthcare costs associated with NCDs, particularly among vulnerable populations [31]. Additionally, the burden of NCDs is higher among women than among men [31]. This disparity is further reinforced by limited access to healthcare, health information, and education, as well as limited control over income and decision-making among women [31]. Women also frequently serve as caregivers for family members with NCDs, thereby reducing their income and limiting their opportunities [31]. Taken together, these factors increase women's vulnerability and worsen their financial risks [31]. Achieving the targets outlined in this action plan may indirectly reduce women's vulnerability and enhance their contribution to the economy, thereby contributing to the achievement of SDG 5 (gender equality) [32]. Furthermore, evidence suggests that properly designed wellness programs and healthy workplaces improve people's well-being and promote healthy working lives, ultimately leading to higher productivity [31]. Adoption of this action plan could therefore increase economic productivity by reducing NCDs and improving overall well-being. 

There are some suggestions regarding the negative unintended consequences of the action plan. For example, tobacco control policies emphasize the creation of smoke-free environments to reduce exposure to second-hand smoke (SHS) [3]. Some concerns have been raised that this could increase poor air quality in homes and indoor SHS exposure, particularly among children and pregnant women, because individuals who are deterred from smoking in public places may instead smoke at home [33]. Furthermore, as argued by Bauld [34], it has been theorized that this negative outcome may be more common among individuals from low-income socioeconomic backgrounds. Considering these views, one could suggest that smoke-free environments may inadvertently contribute to socioeconomic disparities because increased exposure to SHS among low-income populations could lead to a higher burden of chronic disease [35]. Consequently, this could result in higher healthcare costs and increased poverty within this socioeconomic group [35]. Additionally, although increasing tobacco and alcohol taxes is considered one of the most effective approaches to reducing the consumption of tobacco and alcohol products, some evidence suggests that higher taxes may contribute to an increase in illicit trade [36-38].

The evidence presented against smoke-free environments and tobacco taxation, however, is not supported by widespread or robust data. The implementation of laws and legislation, including increased taxation and public smoking bans, is effective in reducing smoking and harmful alcohol use. Consequently, these measures can reduce consumption and improve health outcomes across different socioeconomic groups [39].

*Perspectives* 

NCDs are a global problem that threatens economic development, sustainability, and the achievement of the SDGs [1]. Therefore, the adoption of global resolutions to mitigate this problem is crucial [1]. The Global Action Plan for the Prevention and Control of Noncommunicable Diseases 2013-2020 recognizes this problem and emphasizes the need for collaborative efforts [3]. Its emphasis on collaboration to achieve health outcomes is similar to that of Health 2020 [40]. However, it highlights that a world free of NCDs can only be achieved through collaborative strategies at the national, regional, and international levels [3]. Additionally, because it is a global strategy, the action plan provides a foundation for a broader agenda by establishing a basis upon which other policies can be developed [3]. This action plan is linked to national strategies in areas including health, transport, finance, agriculture, the environment, and economic development [3]. Based on this, adopting the proposed collaborative strategy can help ensure that the established objectives and targets are achieved by creating opportunities for different national sectors to work together. Consequently, this collaboration can contribute to the successful implementation of the action plan.

Table 1 summarizes the findings using the 2008 Davies Provenance Framework.

**Table 1:** Summary of findings using the Davies Provenance Analysis Framework WHO: World Health Organization, UN: United Nations, NCDs: noncommunicable diseases.

Davies framework categories	Summary of findings
Primary purpose	This action plan is aimed at implementing the United Nations political declaration on NCDs and encouraging multilevel and multisectoral collaboration. Also, it emphasizes reducing the burden of NCDs by targeting risk factors like physical inactivity, tobacco use, unhealthy diets, and alcohol
Precedents	It was built upon prior resolutions, including the 2020 Global NCDs plan and the WHO Framework Convention on Tobacco Control. It is underpinned on core principles of universal health coverage, human rights, and social justice
Priorities	The severe health and economic burdens of NCDs created the necessary political urgency. Consistently, merging politics, problems, and policy streams created a window of opportunity for a collaborative global framework
Participants	WHO Member States, United Nations Agencies, private sector, non-governmental organizations, and academia. The WHO championed and coordinated this effort while allowing national governments to lead the implementation, considering their uniqueness. Individual citizens were not directly involved but indirectly through their governments
Process and procedures	It was implemented over a period of seven years (2013-2020), with a total estimated cost of 940.26 million USD. Additionally, structural support and guidance for surveillance, personnel training, and capacity building were provided by the WHO
Practicalities	It is faced with individuals' resistance exacerbated by poverty and structural barriers from competing national priorities. Anticipates resistance from profit-driven private sectors (tobacco and alcohol companies)
Perversities	Supports the achievement of the SDGs and enhances economic productivity. The main unintended negative consequence is that it risks increasing second-hand indoor exposure to tobacco, driving illegal tobacco sale or trade, and causing financial strain on individuals especially those from low-income groups
Perspectives	This global strategy integrates healthcare with other sectors such as transport, agriculture, and finance. It emphasizes the cooperation of the national, regional, and international bodies to reduce the global burden of NCDs

Discussion

By applying the Davies Provenance Framework, this analysis reveals that the WHO Global Action Plan 2013-2020 was designed robustly and a well-precedented policy that merged flexible national implementation strategies with global urgency effectively [3]. It also highlighted that its theoretical effectiveness heavily relies on properly navigating resistance and mitigating unforeseen socioeconomic consequences. 

One of the major enhancers of these unintended consequences, and a potential resistance to the actualization of the global target, is addiction. For example, because of addiction, people who are prevented from smoking in public places smoke indoors, purchase tobacco products regardless of the price, and are marketed to illegal tobacco traders [23]. 

These vulnerabilities (resistance and unintended consequences) were substantiated by experience. For example, halfway evaluation in 2020 showed an alarmingly inadequate progress, and that countries were not on track to meet the predefined targets [41]. As a result, the timeline was extended to 2030 by the WHO, who further established an implementation roadmap for 2023-2030 [41]. According to the WHO analysis, chronic underfunding, lack of adequate political will, and factors outside the healthcare system were the driving forces for low progress in the actualization of the set targets among the member nations 

The integration of leadership and change management models/theories recommends that solving this deep-seated resistance to implementation such as industrial pushback, public addiction, and competing national budgets requires dynamic leadership [25]. A blend of transformational leadership (to inspire behavioral changes and raise awareness) and transactional leadership (to incentivize compliance with the action plan at the governmental level) [25]. 

Limitations

Although the Davies Provenance Framework allows for a systematic and stakeholder-centered study, its limitations should be acknowledged. This framework is not a completely accurate reflection of the complexity and multidimensional nature of public health. By putting emphasis on stakeholders over political differences/dynamics, it risks minimizing the complex economics, geopolitics, and realities that determine the implementation of policies across the WHO Member States. Furthermore, the linear categorization adopted by this framework may largely simplify the deep-rooted interconnectedness of social determinants of health. Thus, by providing a top-down view, it structurally distances the analysis of grassroots, including community-level public health lived realities. 

Nevertheless, it was adopted because it is modeled to allow a methodical study of the call to action from the perspective of the stakeholders and effectively map systematically the organizational drivers and desired outcomes of this action plan [11]. 

## Conclusions

The WHO Global Action Plan for the Prevention and Control of Noncommunicable Diseases 2013-2020 represents a comprehensive framework aimed at addressing the growing global health and economic burden of NCDs through concerted and multisectoral action.

Applying the Davies Provenance Framework to this action plan underscores its strengths, particularly its focus on stakeholder involvement and engagement and its alignment with the SDGs. However, the analysis also revealed vulnerabilities within the policy, particularly regarding behavioral resistance and socioeconomic disparities. Although the action plan accounts for systemic barriers at the theoretical level, its effectiveness in practice is highly dependent on context-specific implementation by Member States. Finally, this analysis suggests that policymakers should proactively mitigate unintended socioeconomic perversities, including indoor tobacco exposure and illicit trade.
